# Sex differences in heart failure patients assessed by combined echocardiographic and cardiopulmonary exercise testing

**DOI:** 10.3389/fcvm.2023.1098395

**Published:** 2023-02-06

**Authors:** Zach Rozenbaum, Yoav Granot, Ben Sadeh, Ofer Havakuk, Joshua H. Arnold, Jason Shimiaie, Michael Ghermezi, Orly Barak, Yanai Ben Gal, Yacov Shacham, Gad Keren, Yan Topilsky, Michal Laufer-Perl

**Affiliations:** ^1^Cardiology Division, Tel Aviv Sourasky Medical Center, Affiliated to the Sackler Faculty of Medicine, Tel Aviv-Yafo, Israel; ^2^Section of Cardiology, Tulane University School of Medicine, New Orleans, LA, United States; ^3^Department of Medicine, University of Illinois at Chicago, Chicago, IL, United States; ^4^Cardiac Surgery Division, Tel Aviv Sourasky Medical Center, Sackler Faculty of Medicine, Tel Aviv-Yafo, Israel

**Keywords:** sex, heart failure, echocardiography, cardiopulmonary exercise, peak VO_2_

## Abstract

**Background:**

We aimed to test the differences in peak VO_2_ between males and females in patients diagnosed with heart failure (HF), using combined stress echocardiography (SE) and cardiopulmonary exercise testing (CPET).

**Methods:**

Patients who underwent CPET and SE for evaluation of dyspnea or exertional intolerance at our institution, between January 2013 and December 2017, were included and retrospectively assessed. Patients were divided into three groups: HF with preserved ejection fraction (HF*p*EF), HF with mildly reduced or reduced ejection fraction (HF*mr*EF/HF*r*EF), and patients without HF (control). These groups were further stratified by sex.

**Results:**

One hundred seventy-eight patients underwent CPET-SE testing, of which 40% were females. Females diagnosed with HF*p*EF showed attenuated increases in end diastolic volume index (*P* = 0.040 for sex × time interaction), significantly elevated E/e' (*P* < 0.001), significantly decreased left ventricle (LV) end diastolic volume:E/e ratio (*P* = 0.040 for sex × time interaction), and lesser increases in A-VO_2_ difference (*P* = 0.003 for sex × time interaction), comparing to males with HF*p*EF. Females diagnosed with HF*mr*EF/HF*r*EF showed diminished increases in end diastolic volume index (*P* = 0.050 for sex × time interaction), mostly after anaerobic threshold was met, comparing to males with HF*mr*EF/HF*r*EF. This resulted in reduced increases in peak stroke volume index (*P* = 0.010 for sex × time interaction) and cardiac output (*P* = 0.050 for sex × time interaction).

**Conclusions:**

Combined CPET-SE testing allows for individualized non-invasive evaluation of exercise physiology stratified by sex. Female patients with HF have lower exercise capacity compared to men with HF. For females diagnosed with HF*p*EF, this was due to poorer LV compliance and attenuated peripheral oxygen extraction, while for females diagnosed with HF*mr*EF/HF*r*EF, this was due to attenuated increase in peak stroke volume and cardiac output. As past studies have shown differences in clinical outcomes between females and males, this study provides an essential understanding of the differences in exercise physiology in HF patients, which may improve patient selection for targeted therapeutics.

## 1. Introduction

In patients with heart failure (HF), the assessment of functional capacity by cardiopulmonary exercise testing (CPET) is used in evaluating physical function and patient prognosis and identifying patients suitable for cardiac transplantation. However, the current cutoffs for transplantation are more representative of male populations and extrapolated to females (1). It is known that peak VO_2_ is lower in healthy females compared to males and potentially much lower for females in the setting of HF and poor ventricular function (2). Due to the under-representation of females in HF trials and in the literature surrounding functional capacity, our understanding of potential sex differences that may exist and that could affect therapy, functional assessment, and recommendations for females with HF, are limited (3).

Any factor that limits peak oxygen consumption (VO_2_) by reducing the rate of O_2_ delivery or its utilization by peripheral tissue can lead to limitations in overall exercise capacity. The physiology of O_2_ delivery and its utilization is determined by a series of steps that ultimately result in aerobic mitochondrial respiration in the peripheral musculature. This cascade includes alveolar ventilation, O_2_ diffusion into plasma and red blood cells, transport of oxygen through the cardiovascular (CV) system by the heart [cardiac output (CO)] and peripheral vessels to the skeletal muscles, and ultimately followed by entry into the mitochondria. Each of these steps in the O_2_ pathway can be quantified by using protocols combining cardiopulmonary stress tests (CPET) with invasive hemodynamic assessment through cardiac catheterization (4). However, this pathway of testing is limited due to its invasive nature and leads to limitations through selection bias and its limited anatomical data.

Previous studies (5, 6) have shown the benefit of combined CPET and stress echo (SE) protocol, allowing non-invasive comprehension of the mechanism of exercise intolerance in patients with HF, and its potential for clinical management. However, there is limited literature evaluating the differences in the mechanism between males and females diagnosed with HF, by using this novel CEPT-SE protocol (5, 6).

Therefore, we aimed to test the mechanisms for differences in peak VO_2_ between males and females diagnosed with HF through the non-invasive CEPT-SE protocol, assessing multiple hemodynamic responses to exercise, in predefined activity levels.

## 2. Methods

### 2.1. Study population

Between January 2013 and December 2017, 248 combined CPET and SE exams were performed using our novel protocol. All patients were clinically stable and ambulatory and referred for the evaluation of effort intolerance or dyspnea. We excluded patients who presented with primary valvular disease (aortic valve replacement *n* = 5, aortic stenosis *n* = 5, rheumatic mitral stenosis *n* = 12, organic mitral regurgitation *n* = 3), hypertrophic cardiomyopathy (*n* = 20), sinoatrial block (*n* = 1), active ischemia (*n* = 2), atrial septal defect (*n* = 1), primary pulmonary hypertension (*n* = 2), and mitochondrial myopathy (*n* = 1), as those disorders might influence the results of the test. Furthermore, none of the patients had congenital heart disease. We also excluded patients who were unable to complete exercise on a semi-recumbent bicycle (respiratory exchange ratio < 1.0; *n* = 15) or were with inadequate acoustic windows (*n* = 3). The remaining 178 patients were divided into three groups (control group, HF*p*EF, HF*mr*EF/HF*r*EF). The rationale for combining patients diagnosed with HF*mr*EF and HF*r*EF into one group was according to the ESC 2021 HF guidelines suggesting those two groups will benefit from similar therapies (7). Each group was divided based on sex [control group; *M* = 23 (61%), *F* = 15 (39%), HF*p*EF; *M* = 43 (55%), *F* = 35 (45%), and HF*mr*EF/HF*r*EF; *M* = 40 (65%), *F* = 22 (35%)]. The control group of patients all had normal exercise capacity and normal baseline echocardiography. Diagnosis of HF*p*EF and HF*mr*EF/HF*r*EF was made prior to testing, and was based on clinical signs and symptoms of HF as defined by the criteria of Rich et al. (8). Diagnosis of HF*p*EF was defined as patients with resting echocardiography ejection fraction EF ≥50%, while HF*mr*EF/HF*r*EF was defined as resting echocardiography with EF < 50%. The retrospective collection and analysis of data was approved by the institutional review board (IRB Committee project approval number 0346-13-TLV).

### 2.2. Sub-group analysis

We have previously shown that effort induced LV and RV, hemodynamic, and peripheral changes differ significantly between patients with normal cardiovascular effort capacity, HF*p*EF, and HF*mr*EF/HF*r*EF (5). Thus, in this study we performed subgroup analyses comparing CV and peripheral responses to exercise in males and females stratified within three groups: control group, patients with HF*p*EF, and patients with HF*mr*EF/HF*r*EF.

### 2.3. Exercise protocol

A symptom-limited graded ramp bicycle exercise test was performed in the semi-supine position on a tilting dedicated microprocessor-controlled eddy current brake stress echo cycle ergo meter (Ergoselect 1000 L, CareFusion, USA). We estimated the expected peak VO_2_ max based on the patient's age, height, and weight, while also including the patient's history. We then calculated the work rate increment necessary to reach each individual patient's estimated peak VO_2_ in 8–12 min. The protocol included 3 min of unloaded pedaling, a symptom limited ramp graded exercise, and 2 min of recovery. Breath-by-breath minute ventilation (VE), carbon dioxide production (VCO_2_), and VO_2_ were measured using a Medical Graphics metabolic cart (ZAN, nSpire Health Inc, Germany). Peak VO_2_ was the highest averaged 30-s VO_2_ during exercise (9). Anaerobic threshold was determined manually using the modified V-slope method. VE/VCO_2_ was defined as the lowest immediately after anaerobic threshold, and was expressed as absolute nadir VE/VCO_2_ (9, 10). A 12-lead electrocardiogram (ECG) and non-invasive arterial saturation were monitored continuously, heart rate and blood pressure were measured at rest and every minute during exercise.

### 2.4. Exercise echocardiography testing

Echocardiography images were obtained concurrently with breath-by-breath gas exchange measurements at rest, immediately upon reaching anaerobic threshold, and at maximal exercise capacity. Data collected at each time period included left ventricle (LV) end diastolic volume (LVEDV), end systolic volume (LVESV), EF, stroke volume (SV), Peak E- and A-wave velocities, E wave deceleration time (DT), and e′ in the septal mitral annulus. LVEDV, LVESD, and EF were calculated based on the single plane ellipsoid apical 4 chamber area-length method (11). SV was calculated by multiplying the LV outflow tract area at rest by the LV outflow tract velocity–time integral measured by pulsed-wave Doppler during each activity levels. E/e' ratio was calculated at all effort stages. During sinus tachycardia whenever merging of mitral E and A velocities occurred, peak E wave velocity, DT, A wave velocity, e' and E/e' ratio were measured by the methods used by Nagueh et al. (12). A-VO_2_ difference was calculated by using the Fick equation as: VO_2_/echo calculated cardiac output at each activity level (5, 10).

### 2.5. Statistical analysis

Descriptive results were expressed as mean ± SD for continuous variables and as percentages for categorical variables. For the analysis of differences in echocardiography and exercise variables between the male and female patients we used ANOVA for continuous, normally distributed, Wilcoxon test for other continuous, and Fisher's exact test or Chi-square test, for categorical variables. We used the repeated measures linear model analysis to define the within-group effect for each parameter over time, the between group differences over time, and the group by time interactions. Using an analysis “sex × time” interaction we referred to the difference between the groups overtime. While “group” tested the difference between the results between the two sexes, and “time” tested the in-group increment differences, “sex × time” compared the temporal change between the groups. This allows comparison of the sex-specific response to exercise for each tested parameter. All computations were performed using JMP statistical software for Windows (Version 13.0; SAS Institute Inc).

## 3. Results

### 3.1. Complete cohort results

Clinical and baseline echocardiography characteristics of our cohort in its entirety and stratified by sex are presented in Table 1. Most baseline LV parameters were larger in male patients compared to females, even following adjustment for body surface area (BSA). Of note, although functional parameters for LV (Cardiac index, EF) and RV (fractional area change) were similar in both sexes, E/e' was significantly higher in female patients compared to males (21.4 ± 15.4 vs. 14.5 ± 10.1, *P* = 0.004). Echocardiographic and combined CPET-SE parameters of the entire cohort, stratifies by sex groups in each of the exercise phases are presented in Table 2. Male patients showed a gradual increase in LVEDV when approaching the anaerobic threshold stage, which, combined with the attenuated change in LVESV, resulted in an early SV increase by ≈25% (mean 88.2 vs. 71.6 ml; *P* < 0.0001). LVEDV and LVESV volume decreased between the anaerobic threshold and maximal exercise phases resulting in a maintained average SV in the final part of the effort. However, in female patients, the gradual increase in LV volumes up to the anaerobic threshold was attenuated (*P* < 0.05 for sex × time interaction) resulting in a trend toward reduction in increases in SV during effort (*P* = 0.09 for sex × time interaction). Most importantly, female patients showed attenuated increases in A-VO_2_ difference (*P* = 0.04 for sex × time interaction), suggesting that the decrease in exercise capacity in females compared to male patients is related in part to peripheral factors.

**Table 1:** Baseline characteristics according to sex.

	**Males**	**Females**	***P*-value**
Age, years	59.3 (±17.1)	61.2 (±13.0)	0.007
Body surface area, m^2^	1.96 (±0.17)	1.75 (±0.19)	<0.001
Heart rate, beats per minute	79.2 (±13.4)	82.3 (±17.1)	0.136
Systolic blood pressure, mmHg	133.3 (±38.5)	140.1 (±26.6)	0.047
Diastolic blood pressure, mmHg	75.1 (±23.1)	80.8 (±12.5)	0.04
Left ventricle end diastolic diameter, mm	51.6 (±8.6)	47.6 (±6.5)	<0.001
Left ventricle end systolic diameter, mm	35.5 (±10.7)	30.3 (±8.7)	<0.001
Left ventricle ejection fraction, %	51.3 (±16.7)	54.1 (±15.1)	0.239
Stroke volume index, ml/m^2^	36.8 (±9.5)	38.6 (±11.0)	0.267
Cardiac index, ml/min/m^2^	2.8 (±0.8)	3.0 (±0.8)	0.100
Left ventricle end diastolic volume index, ml	79.4 (±30.8)	66.6 (±20.4)	0.002
Left ventricle end systolic volume index, ml	40.9 (±26.1)	31.2 (±16.7)	0.005
Left ventricle mass index, gr/m^2^	123 (±39)	103 (±29)	0.002
Left atrial diameter, mm	41.5 (±11.7)	42.8 (±8.9)	0.397
Left atrial volume index, ml/m^2^	38.1 (±18.1)	44.8 (±23.1)	0.060
E wave cm/s base	78.5 (±30.1)	107.6 (±55.7)	<0.001
A wave cm/s base	60.5 (±27.7)	73.1 (±40.6)	0.060
E/A ratio	1.4 (±0.6)	1.4 (±0.6)	0.168
Deceleration time, ms	216 (±89)	286 (±177)	0.006
E' cm/s	6.2 (±2.5)	5.9 (±2.3)	0.509
E/e'	14.5 (±10.1)	21.4 (±15.4)	0.004
Systolic pulmonary artery pressure, mmHg	33.3 (±10.8)	36.0 (±12.7)	0.397
S wave, cm/s	5.6 (±2.1)	4.9 (±1.4)	0.003
Right atrial area, cm^2^	17.8 (±6.1)	15.6 (±5.1)	0.01
Right ventricle end diastolic area, cm^2^	24.5 (±6.9)	20.3 (±5.1)	<0.001
Right ventricle end systolic area, cm^2^	14.7 (±5.1)	12.1 (±4.1)	0.001
Right ventricle fractional area change, %	0.39 (±0.1)	0.41 (±0.13)	0.789
FEV1, % predicted	87.4 (±17.7)	85.3 (±19.1)	0.062
FVC, % predicted	82.6 (±17.5)	82.1 (±18.6)	0.689
FEV1-FVC, % predicted	109.2 (±16.6)	106.9 (±11.4)	0.020
Peak VO_2_	1.6 (±0.7)	1.0 (±0.4)	<0.001
Peak VO_2_/kg	19.3 (±9.1)	13.9 (±5.6)	<0.001

FEV1, forced expiratory volume in one second; FVC, forced vital capacity; VO_2_, rate of oxygen consumption.

**Table 2:** Echocardiographic and combined cardiopulmonary exercise- stress echocardiography parameters of the entire cohort according to sex and stratified to the exercise phase.

**Measurement**		**Baseline**	**Anaerobic** **threshold**	**Maximal** **effort**	**Group**	**Time**	Time*group **interaction**
End diastolic volume, ml	Male	155.3 (±64.5)	172.1 (±71.6)	159.2 (±60.2)	<0.001	0.010	0.050
	Female	114.0 (±34.0)	125.0 (±44.4)	115.7 (±40.3)			
End diastolic volume index	Male	79.4 (±30.8)	87.7 (±34.3)	81.6 (±29.7)	0.010	0.020	0.030
	Female	40.9 (±26.1)	71.8 (±25.3)	66.7 (±24.1)			
End systolic volume, ml	Male	80.3 (±52.1)	84.6 (±48.4)	78.0 (±53.7)	0.005	0.050	0.237
	Female	53.8 (±30.6)	52.0 (±31.1)	53.2 (±32.2)			
Ejection fraction, %	Male	51.3 (±16.7)	53.9 (±19.8)	53.4 (±18.4)	0.974	0.070	0.974
	Female	54.1 (±15.1)	58.8 (±15.4)	54.5 (±18.9)			
Tissue Doppler RV S', cm/s	Male	5.6 (±2.1)	7.3 (±2.8)	7.5 (±3.1)	0.542	<0.001	0.542
	Female	4.9 (±1.4)	6.7 (±2.4)	6.6 (±2.7)			
Right ventricle end diastolic area, cm^2^	Male	24.5 (±6.9)	26.6 (±5.8)	25.1 (±6.1)	<0.001	0.020	0.081
	Female	20.3 (±5.1)	21.5 (±4.0)	22.3 (±4.4)			
Right ventricle end systolic area, cm^2^	Male	14.7 (±5.1)	15.6 (±5.0)	14.9 (±5.4)	0.004	0.164	0.545
	Female	12.1 (±4.1)	12.9 (±3.9)	12.8 (±4.5)			
Right ventricle fractional area change, %	Male	0.39 (±0.1)	0.41 (±0.12)	0.40 (±0.15)	0.138	0.572	0.138
	Female	0.41 (±0.13)	0.40 (±0.15)	0.43 (±0.15)			
Tissue Doppler e', cm/s	Male	6.2 (±2.5)	10.2 (±4.8)	11.4 (±6.4)	0.818	<0.001	0.818
	Female	5.9 (±2.3)	8.8 (±4.7)	9.2 (±4.9)			
E/e'	Male	14.5 (±10.1)	13.8 (±9.4)	15.4 (±10.4)	0.001	0.714	0.295
	Female	21.4 (±15.4)	22.3 (±17.1)	22.3 (±16.2)			
Stroke volume, ml	Male	71.6 (±18.9)	88.2 (±27.4)	84.5 (±29.0)	0.030	<0.001	0.090
	Female	65.9 (±16.4)	80.9 (±21.4)	68.9 (±26.7)			
Stroke volume index, ml/m	Male	36.8 (±9.5)	45.2 (±14.4)	43.9 (±14.8)	0.038	<0.001	0.040
	Female	38.6 (±11.0)	46.6 (±12.9)	39.5 (±14.6)			
Heart rate, BPM	Male	79.2 (±13)	107 (±21)	127 (±15)	0.963	<0.001	0.963
	Female	82.3 (±17)	108 (±23)	123 (31)			
Cardiac output, L/min	Male	5.5 (±1.6)	9.3 (±3.4)	10.8 (±4.7)	0.100	<0.001	0.362
	Female	5.3 (±1.4)	9.0 (±2.9)	8.8 (±4.0)			
VO_2_, L/min	Male	0.4 (±0.1)	1.1 (±0.4)	1.6 (±0.7)	<0.001	<0.001	<0.001
	Female	0.3 (±0.1)	0.8 (±0.3)	1.0 (±0.4)			
A-VO_2_ diff, L/L	Male	0.08 (±0.03)	0.11 (±0.03)	0.15 (±0.05)	0.002	<0.001	0.040
	Female	0.07 (±0.02)	0.09 (±0.03)	0.13 (±0.06)			

BPM, beat per minute; VO_2_, rate of oxygen consumption.

### 3.2. Cardiovascular and peripheral responses in the control group

**Figure 1:**
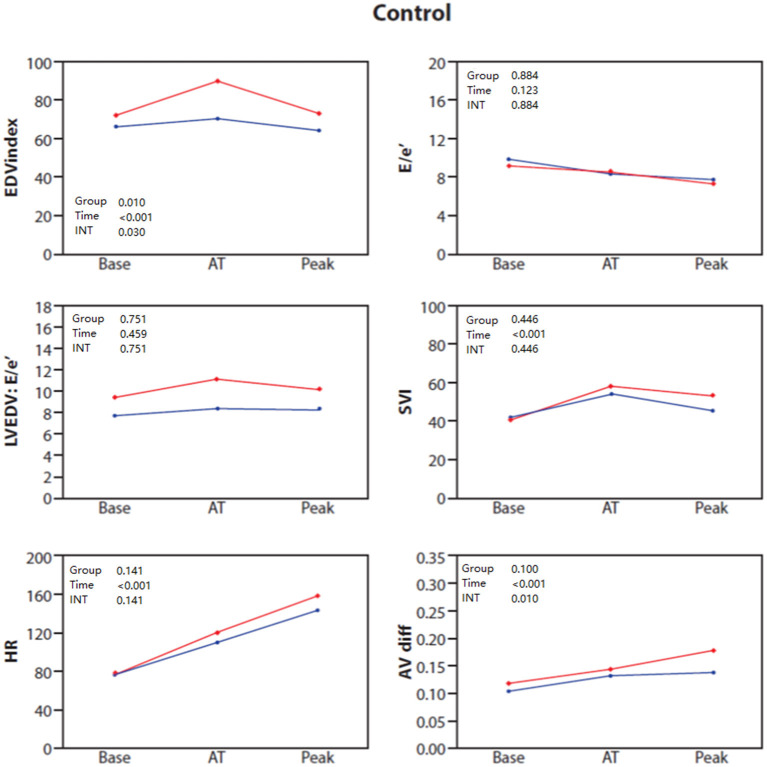
Baseline, anaerobic threshold (AT), and maximal cardiopulmonary exercise (Peak) test and stress echocardiography test for End diastolic volume (EDV) index, E/e', LVEDV: E/e' ratio, stroke volume index (SVI), heart rate (HR) and AVO_2_ difference in control patients stratified by sex: females (blue) and males (red).

Clinical and baseline echocardiographic characteristics of the control group stratified by sex are shown in Supplementary Table 1. Echocardiographic and combined CPET-SE parameters for each sex during each of the exercise phases in the control group are presented in Table 3 and Figure 1. Female patients showed diminished increases in LV volumes when approaching anaerobic threshold (*P* = 0.033 for sex × time interaction), and in VO_2_ and A-VO_2_ difference at peak exercise (*P* < 0.001 and *P* = 0.010, respectively for sex × time interaction), when compared to males in the control group.

**Table 3:** Echocardiographic and combined cardiopulmonary exercise- stress echocardiography parameters in the control group according to sex and stratified to the exercise phase.

**Measurement control**		**Baseline**	**Anaerobic threshold**	**Maximal effort**	**Group**	**Time**	Time*group **interaction**
End diastolic volume, ml	Males	161.7 (±72.7)	187.0 (±69.3)	158.8 (±58.2)	0.033	0.020	0.033
	Females	106.7 (±17.9)	123.0 (±24.8)	107.4 (±29.1)			
End diastolic volume index	Males	72.3 (±17.9)	90.3 (±25.4)	73.8 (±20.0)	0.010	<0.001	0.030
	Females	66.5 (±14.2)	71.9 (±17.8)	64.0 (±21.7)			
End systolic volume, ml	Males	78.3 (±54)	70.5 (±52.8)	68.1 (±49.3)	0.944	0.050	0.944
	Females	42.5 (±18.4)	40.6 (±12.5)	34.2 (±20.2)			
Ejection fraction, %	Males	64.0 (±16.1)	64.8 (±14.1)	66.7 (±14.8)	0.865	0.010	0.865
	Females	61.0 (±13.4)	65.4 (±13.6)	67.3 (±19.0)			
Tissue Doppler RV S', cm/s	Males	7.1 (±1.5)	10.2 (±2.6)	10.6 (±3.2)	0.913	<0.001	0.913
	Females	6.1 (±1.2)	9.0 (±2.5)	8.8 (±2.4)			
Right ventricle end diastolic area, cm^2^	Males	26.5 (±7.3)	28.4 (±5.9)	23.0 (±4.8)	0.020	0.786	0.291
	Females	21.3 (±5.5)	21.2 (±2.9)	22.7 (±4.1)			
Right ventricle end systolic area, cm^2^	Males	15.2 (±3.8)	14.3 (±4.1)	13.6 (±3.9)	0.030	0.070	0.547
	Females	12.8 (±5.2)	10.8 (±2.8)	10.9 (±4.5)			
Right ventricle fractional area change, %	Males	0.4 (±0.09)	0.49 (±0.1)	0.41 (±0.1)	0.191	0.001	0.191
	Females	0.4 (±0.1)	0.49 (±0.1)	0.51 (±0.2)			
Tissue Doppler e', cm/s	Males	8.2 (±2.7)	14.9 (±4.8)	16.5 (±7.2)	0.281	<0.001	0.281
	Females	8.0 (±2.8)	10.9 (±4.7)	14.8 (±3.9)			
E/e'	Males	9.1 (±4.2)	8.6 (±3.9)	7.4 (±3.3)	0.884	0.123	0.884
	Females	9.3 (±4.0)	8.5 (±4.3)	7.7 (±3.0)			
LVEDVi:E/e'	Males	9.5 (±3.0)	11.2 (±3.4)	10.2 (±2.7)	0.751	0.459	0.751
	Females	7.8 (±5.0)	8.5 (±2.9)	8.3 (±2.5)			
Stroke volume, ml	Males	76.2 (±20.4)	108.7 (±28.3)	102.7 (±26.8)	0.100	<0.001	0.296
	Females	71.4 (±12.0)	94.6 (±15.2)	88.9 (±26.4)			
Stroke volume index, ml/m	Males	41.3 (±11.0)	58.5 (±14.0)	53.6 (±14.4)	0.446	<0.001	0.446
	Females	42.6 (±9.2)	54.9 (±11.3)	46.0 (±17.0)			
Heart rate, BPM	Males	76.9 (±19.7)	122.9 (±19.7)	159.6 (±25.1)	0.141	<0.001	0.141
	Females	77.1 (±16.6)	113.1 (±16.6)	145.0 (±20.0)			
Cardiac output, L/min	Males	5.7 (±1.8)	13.2 (±4.0)	16.0 (±5.0)	0.100	<0.001	0.102
	Females	5.6 (±1.1)	11.0 (±2.5)	12.6 (±4.2)			
VO_2_, L/min	Males	0.4 (±0.1)	1.4 (±0.5)	2.5 (±0.78)	<0.001	<0.001	<0.001
	Females	0.3 (±0.07)	1.0 (±0.42)	1.4 (±0.65)			
A- VO_2_ Diff, L/L	Males	0.08 (±0.03)	0.11 (±0.04)	0.16 (±0.04)	0.100	<0.001	0.010
	Females	0.06 (±0.01)	0.09 (±0.02)	0.10 (±0.02)			

RV, right ventricle; LVEDVi, left ventricle end diastolic volume index; BPM, beat per minute; VO_2_, rate of oxygen consumption.

### 3.3. Cardiovascular and peripheral responses in Patients with HF*p*EF

**Figure 2:**
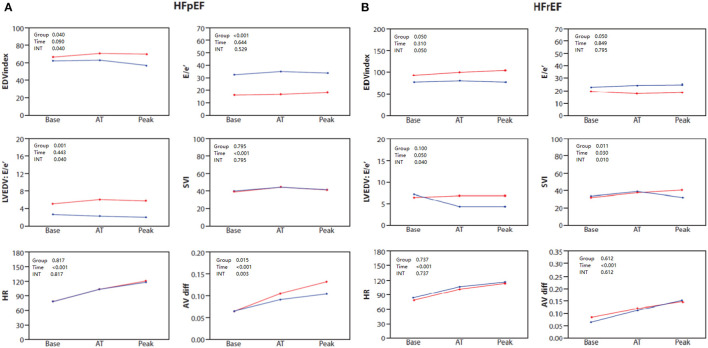
**(A)** Baseline, anaerobic threshold (AT), and maximal cardiopulmonary exercise (Peak) test and stress echocardiography test for End diastolic volume (EDV) index, E/e', LVEDV: E/e' ratio, stroke volume index (SVI), heart rate (HR) and AVO_2_ difference in HF*p*EF patients stratified by sex: females (blue) and males (red). **(B)** Baseline, anaerobic threshold (AT), and maximal cardiopulmonary exercise (Peak) test and stress echocardiography test for End diastolic volume (EDV) index, E/e', LVEDV: E/e' ratio, stroke volume index (SVI), heart rate (HR) and AVO_2_ difference in HF*mr*EF/HF*r*EF patients stratified by sex: females (blue) and males (red).

Clinical and baseline echocardiographic characteristics for HF*p*EF patients stratified by sex are shown in Supplementary Table 1. Echocardiographic and combined CPET-SE parameters for the sex groups in each of the exercise phases in the HF*p*EF group are presented in Table 4 and Figure 2A. The main differences are summarized by three main points. First, female patients showed attenuated increases in LVEDV index (*P* = 0.04 for sex × time interaction), but showed no difference in SV index or CO, compared to males. Second, female patients with HF*p*EF had a significantly increased measured E/e' throughout the exercise protocol (*P* < 0.001) and LVEDV:E/e ratio (*P* = 0.001), compared to males with HF*p*EF. Third female patients with HF*p*EF had significantly diminished increases in A-VO_2_ difference (*P* = 0.003 for sex × time interaction), compared to males with HF*p*EF.

**Table 4:** Echocardiographic and combined cardiopulmonary exercise- stress echocardiography parameters in patients with heart failure and preserved ejection fraction according to sex and stratified to the exercise phase.

**Measurement control**		**Baseline**	**Anaerobic threshold**	**Maximal effort**	**Group**	**Time**	Time*group **interaction**
End diastolic volume, ml	Males	123.1 (±35.4)	137.8 (±32.1)	129.0 (±42.4)	<0.001	0.090	0.050
	Females	103.9 (±22.5)	107.9 (±32.3)	99.6 (±26.7)			
End diastolic volume index	Males	66.9 (±17.5)	70.8 (±16.8)	70.6 (±20.3)	0.040	0.090	0.040
	Females	62.8 (±14.5)	63.1 (±17.0)	57.7 (±15.0)			
End systolic volume, ml	Males	43.9 (±18.5)	51.7 (±27.0)	48.4 (±25.9)	0.010	0.060	0.173
	Females	37.6 (±12.5)	39.0 (±15.2)	35.6 (±14.2)			
Ejection fraction, %	Males	64.6 (±9.6)	63.3 (±15.4)	62.2 (±16.2)	0.563	0.227	0.563
	Females	63.9 (±8.6)	62.4 (±14.7)	63.3 (±14.5)			
Tissue Doppler RV S', cm/s	Males	5.6 (±1.9)	7.3 (±2.3)	7.6 (±2.6)	0.066	<0.001	0.066
	Females	4.9 (±1.5)	6.5 (±2.1)	6.7 (±2.6)			
Right ventricle end diastolic area, cm^2^	Males	21.1 (±5.0)	24.5 (±4.5)	24.6 (±5.1)	<0.001	0.010	0.276
	Females	18.6 (±3.6)	20.8 (±4.2)	20.5 (±4.4)			
Right ventricle end systolic area, cm^2^	Males	12.3 (±3.4)	13.2 (±3.7)	12.8 (±4.1)	0.050	0.199	0.383
	Females	10.6 (±2.9)	11.6 (±3.1)	10.8 (±3.7)			
Right ventricle fractional area change, %	Males	0.42 (±0.11)	0.47 (±0.11)	0.48 (±0.14)	0.383	0.050	0.383
	Females	0.43 (±0.12)	0.44 (±0.14)	0.48 (±0.11)			
Tissue Doppler e', cm/s	Males	5.9 (±2.1)	8.5 (±3.8)	9.9 (±4.9)	0.086	<0.001	0.086
	Females	5.7 (±2.5)	8.6 (±4.9)	9.1 (±5.4)			
E/e'	Males	16.6 (±3.9)	17.4 (±11.5)	18.8 (±15.7)	<0.001	0.644	0.529
	Females	30.4 (±11.8)	35.0 (±19.0)	34.1 (±17.7)			
Left ventricle end diastolic volume index: E/e'	Males	5.2 (±6.1)	6.1 (±7.6)	5.9 (±7.1)	0.001	0.443	0.040
	Females	2.6 (±4.6)	2.3 (±6.3)	2.0 (±5.3)			
Stroke volume, ml	Males	74.8 (±14.5)	88.1 (±18.7)	82.2 (±18.2)	0.010	<0.001	0.010
	Females	65.5 (±15.5)	79.5 (±18.6)	71.2 (±22.7)			
Stroke volume index, ml/m	Males	38.7 (±7.5)	44.6 (±11.0)	41.8 (±10.2)	0.795	<0.001	0.795
	Females	40.2 (±10.1)	45.2 (±11.4)	41.3 (±12.5)			
Heart rate, BPM	Males	78.7 (±13.7)	104.1 (±20.5)	121.3 (±27.1)	0.817	<0.001	0.817
	Females	78.9 (±15.0)	104.8 (±23.0)	118.8 (±30.5)			
Cardiac output, L/min	Males	5.8 (±1.3)	8.9 (±2.8)	9.7 (±2.8)	0.100	<0.001	0.449
	Females	5.1 (±1.5)	8.7 (±2.6)	8.9 (±3.5)			
VO_2_, L/min	Males	0.39 (±0.13)	0.95 (±0.28)	1.29 (±0.4)	<0.001	<0.001	0.010
	Females	0.33 (±0.07)	0.75 (±0.25)	0.95 (±0.36)			
A-VO_2_ Diff, L/L	Males	0.07 (±0.02)	0.11 (±0.03)	0.13 (±0.04)	0.015	<0.001	0.003
	Females	0.07 (±0.02)	0.09 (±0.02)	0.10 (±0.05)			

RV, right ventricle; BPM, beat per minute; VO_2_, rate of oxygen consumption.

### 3.4. Cardiovascular and peripheral responses in patients with HF*mr*EF/HF*r*EF

Clinical and baseline echocardiographic characteristics of the patients with HF*mr*EF/HF*r*EF stratified by sex are shown in Supplementary Table 1. Echocardiographic and combined CPET-SE parameters of the sex groups in each of the exercise phases of HF*mr*EF/HF*r*EF patients are presented in Table 5 and Figure 2B. We found the main differences between the sexes in HF*mr*EF/HF*r*EF patients to be that females demonstrated attenuated increases in LVEDV (*P* = 0.05 for sex × time interaction), which resulted in a diminished increase in SV index (*P* = 0.010 for sex × time interaction) and CO (*P* = 0.05 for sex x time interaction), compared to males with HF*mr*EF/HF*r*EF. Although there were no significant differences in E/e', the LVEDV:E/e ratio was lower in the late stages of exercise in females. Interestingly, there was no difference between sexes in A-VO_2_ throughout the exercise protocol.

**Table 5:** Echocardiographic and combined cardiopulmonary exercise- stress echocardiography parameters in patients with heart failure with mildly reduced and reduced ejection fraction according to sex and stratified to the exercise phase.

**Measurement control**		**Baseline**	**Anaerobic threshold**	**Maximal effort**	**Group**	**Time**	Time*group **interaction**
End diastolic volume, ml	Male	181.9 (±72.9)	206.7 (±91.8)	193.4 (±61.4)	0.010	0.286	0.050
	Female	125.6 (±47.3)	141.2 (±65.2)	131.2 (±52.8)			
End diastolic volume index	Male	93.6 (±32.5)	100.7 (±41.2)	105.0 (±30.1)	0.050	0.31	0.050
	Female	77.7 (±24.4)	81.7 (±37.5)	78.7 (±29.5)			
End systolic volume, ml	Male	116.9 (±53.8)	130.6 (±88.0)	114.0 (±60.2)	0.050	0.733	0.801
	Female	79.3 (±34.4)	78.4 (±43.9)	82.7 (±34.7)			
Ejection fraction, %	Male	36.4 (±9.1)	39.1 (±18.1)	42.9 (±14.9)	0.356	0.050	0.356
	Female	37.3 (±8.9)	40.3 (±13.9)	36.9 (±11.3)			
Tissue Doppler RV S', cm/s	Male	5.2 (±2.1)	5.9 (±2.2)	5.8 (±2.6)	0.201	0.010	0.201
	Female	4.5 (±1.2)	5.2 (±1.2)	5.7 (±2.2)			
Right ventricle end diastolic area, cm^2^	Male	27.0 (±7.6)	28.9 (±6.2)	27.4 (±7.3)	0.004	0.817	0.050
	Female	20.7 (±5.8)	21.3 (±4.8)	23.7 (±4.6)			
Right ventricle end systolic area, cm^2^	Male	17.0 (±6.2)	19.4 (±5.0)	17.8 (±6.2)	0.080	0.100	0.100
	Female	13.4 (±4.3)	15.7 (±3.9)	16.2 (±3.1)			
Right ventricle fractional area change, %	Male	0.36 (±0.1)	0.32 (±0.1)	0.33 (±0.1)	0.449	0.009	0.449
	Female	0.35 (±0.1)	0.26 (±0.1)	0.31 (±0.1)			
Tissue Doppler e', cm/s	Male	5.5 (±2.3)	8.8 (±4.3)	8.7 (±4.3)	0.646	0.003	0.646
	Female	5.3 (±1.7)	6.8 (±3.1)	8.0 (±3.9)			
E/e'	Male	18.2 (±12.7)	16.7 (±11.6)	17.9 (±9.9)	0.050	0.849	0.795
	Female	21.6 (±13.9)	22.6 (±13.9)	22.8 (±14.)			
Left ventricle end diastolic volume index:E/e'	Male	6.6 (±3.4)	6.9 (±6.5)	6.9 (±4.0)	0.100	0.050	0.040
	Female	7.2 (±4.8)	4.4 (±2.4)	4.3 (±3.4)			
Stroke volume, ml	Male	65.9 (±19.8)	77.7 (±26.)	81.6 (±35.0)	0.036	0.050	0.030
	Female	63.6 (±17.5)	70.4 (±26.2)	62.5 (±21.2)			
Stroke volume index, ml/m	Male	33.2 (±9.1)	39.0 (±14.5)	41.7 (±17.0)	0.011	0.030	0.010
	Female	35.1 (±10.5)	39.6 (±11.5)	32.7 (±11.1)			
Heart rate, BPM	Male	79.0 (±14.7)	102.0 (±21.2)	114.6 (±28.9)	0.737	<0.001	0.737
	Female	84.1 (±19.2)	106.5 (±19.9)	115.2 (±26.1)			
Cardiac output, L/min	Male	5.1 (±1.6)	8.1 (±3.0)	9.4 (±4.6)	0.058	<0.001	0.050
	Female	5.1 (±1.2)	7.7 (±3.2)	7.0 (±2.7)			
VO_2_, L/min	Male	0.4 (±0.1)	0.9 (±0.31)	1.2 (±0.47)	<0.001	<0.001	0.007
	Female	0.3 (±0.08)	0.7 (±0.2)	0.8 (±0.24)			
A-VO_2_ Diff, L/L	Male	0.09 (±0.02)	0.12 (±0.03)	0.15 (±0.05)	0.612	<0.001	0.612
	Female	0.07 (±0.02)	0.11 (±0.04)	0.14 (±0.06)			

RV, right ventricle; BPM, beat per minute; VO_2_, rate of oxygen consumption.

## 4. Discussion

Our major findings are that: (1) the described combined CPET-SE protocol allows for detailed individualized non-invasive evaluation of exercise physiology throughout varying levels of effort for male and female patients; (2) Female patients with HF*p*EF have lower exercise capacity when compared with similar male patients due to poorer LV compliance, higher filling pressures, and attenuated peripheral oxygen extraction; and (3) Female patients with HF*mr*EF/HF*r*EF have lower exercise capacity when compared with similar male patients due to diminished increases in LV volumes at later stages of exercise induced stress resulting in attenuated increases in SV and CO.

Despite the fact that females represent around 50% of the total population of patients diagnosed with HF (13), they have been under-represented in currently published HF studies (14). CPET, with the measurement of peak VO_2_, has become the cornerstone tool for assessing functional capacity and predicting outcomes in patients with HF (8, 15). Surprisingly, the under-representation of females in CPET studies is even more apparent (15–17). These gaps in information limit our understanding of risk stratification, recommendations for physical therapy, and advanced HF intervention in females. Given the limited available data in CPET parameters for the clinical assessment of females with HF, we have devised our new combined CPET and SE protocol (5), which enables clinicians to non-invasively assess multiple responses and parameters related to dynamic exercise, and to define the mechanisms for difference in peak VO_2_ between male and female patients diagnosed with HF*p*EF or HF*mr*EF/HF*r*EF.

### 4.1. HF*p*EF

The main differences found between female and male patients with HF*p*EF were that female patients showed attenuated increases in LVEDV, increased E/e', and decreases in LVEDV:E/e ratio throughout the protocol. E/e' is the echocardiographic correlate of LV diastolic pressure and was higher in female patients throughout exercise. We have previously shown that LVEDV:E/e' ratio is an estimate of LV compliance (5) and estimates one's ability to utilize the Frank-Starling mechanism. Decreased LVEDV:E/e' ratio represent the failure of LV to appropriately increase its size proportionately to filling pressure. In this present study, we show that LVEDV:E/e' ratio was lower in female patients with HFpEF. This finding suggests that diastolic dysfunction assumes a role in the mechanism of exercise intolerance of female patients with HFpEF. The reduced ability of the stiffened LV to increase its size despite elevated filling pressure is exaggerated. The result is high pressure but relatively low volume, which in turn not only decreases SV but may also negatively impact LV contractility through the Frank-Starling mechanism. Interestingly, a recent hemodynamic study has shown that females with HFpEF exhibit a larger increase in pulmonary capillary wedge pressure in response to rapid saline loading compared to men (18). Another community-based study has shown greater age-related increase in LV stiffness in females compared to males, in concordance with our results (19, 20). Increases in LV diastolic stiffness elevate chamber filling pressures at similar chamber volumes, which may contribute to the lower effort capacity in female patients with HFpEF. Another important sex related difference in the HF*p*EF group was the diminished increases in A-VO_2_ difference likely suggesting the presence of less skeletal muscle mass or lower capillary to muscle fiber ratio (21). It may also suggest that lower metabolic efficiency in females with HF*p*EF contributes to their reduced effort capacity.

### 4.2. HF*mr*EF/HF*r*EF

The main sex related differences that we found between HF*mr*EF/HF*r*EF patients suggest that female patients demonstrated attenuated increases in LVEDV, which resulted in diminished increases in SV and CO. It is generally accepted that in healthy individuals during incremental exercise SV increases and plateaus at ~50% of VO_2_max mainly due to decreased diastolic filling time. Incremental exercise SV response of plateau with a drop has also been described (22). Attenuated increases in LVEDV may occur by decreased diastolic filling time as well as diastolic dysfunction and may result in a less effective Frank-Starling mechanism and subsequently decreased SV. Interestingly, although LV compliance was similar between males and females at the initiation of exercise, compliance lessened only in female patients upon reaching the anaerobic phase (Figures 2A, B), further reaching its nadir at peak exercise. This stress related reduction in compliance may be related to the innate load dependence physiology of LV compliance. LV stiffness may increase in proportion to filling volume changes and can be likened to the stiffness of a balloon, in which the amount of pressure required to cause a given increase in volume increases as the volume of the balloon is increased (5). The interesting disparity between females and males in this load dependent reduction in compliance may be related to the greater degree of concentric remodeling and LV diastolic elastance found in females (23). As explained by LaPlace law wall tension is maintained by the relation between pressure and radius, or left ventricle end-diastolic pressure (LVEDP) and LVEDV. A decrease in chamber size due to concentric remodeling, or an attenuated increase in LVEDV, may result in an increased LVEDP, thereby increasing LA pressure and contribute to exercise intolerance.

### 4.3. Combined cardio-pulmonary and echo exercise protocol

Prior cardiopulmonary exercise studies aimed at examining the determinants of exercise performance in females have mainly used protocols based on invasive tools for the assessment of CO. These studies are inherently limited by their invasive nature, which thereby limits their general clinical applicability, and may lead to selection bias (4, 24–26). A major strength of our protocol is the allowance for the simultaneous recording of CPET (peak VO_2_ VE/VCO_2_), echo-derived parameters (SV, CO, left and right ventricular function, filling pressures), and combined parameters (A-VO_2_ difference), which provides clinicians with a wealth of clinically significant, actionable information regarding individualized patient cardiovascular physiology summarized in a singular panel at the conclusion of one exam. Our study has several other advantages when compared to previous studies that used combined CPET and echocardiography methodology (27–30). First, we did not use a predetermined increase in work, we instead elected to calculate the expected work in watts after recording the height, weight, age, sex, and medical history of our patients. We then calculated the work rate increment necessary to reach the patient's estimated peak work in 8–12 min. The extended period of effort permitted the acquisition of comprehensive hemodynamic data (mitral inflow velocities, tissue Doppler annular signals, outflow tract velocity integral) in addition to LV volumes, thus, uniquely allowing for the calculation of LV compliance. Second, we obtained echocardiography images at individual stages of effort (rest, anaerobic threshold, maximal) instead of preselected power outputs, which may represent different stages of effort in different patients. Third, we did not stop β-blocker therapy prior to examination as we sought to determine the individual factors responsible for effort intolerance in the setting of “real-life” medical management, which is much more applicable and may allow for the administration of specific recommendations in individual subjects.

## 5. Limitations

Our study has several limitations. First, it is a single center study and thus generalization of our results is limited. Second, it is a retrospective study, and therefore our results are subject to the effects of possible confounders and may be biased by the nature of this design. Third, our imaging protocol was performed in the semi-supine position, which generates a somewhat different hemodynamic response than the more commonly used treadmill exercise. Last, SV and CO measurements may have been underestimated or overestimated because of the technical challenge of acquiring echocardiographic images during exercise. However, this technique has been used successfully and validated against radionuclide angiography and Fick SV with reported excellent day-to-day reproducibility and intraobserver and interobserver variability (28).

## 6. Conclusion

Based on previous evidence regarding the role of CPET in HF patients, current position papers (14) advocate for three ‘cut-point’ values (>18, 18–10, or <10 ml/kg/min) of peak VO_2_ for prognostication and risk stratification in patients with HF. However, there is still little data to aid in stratifying several large and clinically significant subgroups of patients: particularly females, or patients with HF*p*EF. Peak VO_2_ is significantly lower in females when compared to males with similar ventricular function. Our data suggest that the decrease in exercise capacity female HF (either HF*mr*EF/HF*r*EF or HF*p*EF) patients experience compared to male patients is related to a sex specific impairment in LV compliance. However, while in HF*mr*EF/HF*r*EF, the reduced compliance results in decreases in overall CO, in HF*p*EF, it results in elevated LV filling pressures without significant changes in SV. Another important difference is that in female patients with HF*p*EF, peripheral factors contribute significantly to the reduction in measured effort capacity, while in HF*mr*EF/HF*r*EF they do not. The acquisition of hemodynamic data obtained non-invasively by combined stress echocardiography and CPET improves clinician ability to measure comprehensive cardiac and respiratory function and may aid significantly in therapeutic decision-making. Furthermore, we believe that the clearer understanding of differences in exercise hemodynamics and peak oxygen consumption afforded by our new protocol may reveal differing sex-specific diagnostic strategies in males and females. However, further research is necessary to assess the potential advantages of these potential sex-specific approaches, which can lead to the development of new therapeutic targets that may reveal themselves following further elucidation of the underlying sex differences in myocardial structure and systemic vascular function.

## Data availability statement

The original contributions presented in the study are included in the article/Supplementary material, further inquiries can be directed to the corresponding author.

## Ethics statement

The studies involving human participants were reviewed and approved by Tel Aviv Sourasky 0346-13-TLV. Written informed consent for participation was not required for this study in accordance with the national legislation and the institutional requirements.

## Author contributions

ZR: contributed to conception and design of the work and acquisition, analysis and interpretation of data for the work, and drafting the work and revising it critically for important intellectual content. YG, BS, OH, JA, JS, MG, OB, YB, YS, and YT: contributed to acquisition and interpretation of data for the work and revising it critically for important intellectual content. GK: contributed to conception and design of the work and interpretation of data for the work and revising it critically for important intellectual content. ML-P: contributed to conception and design of the work and acquisition and drafting the work and revising it critically for important intellectual content. All authors contributed to the article and approved the submitted version.
