# Supplementary material for: Sex differences in heart failure patients assessed by combined echocardiographic and cardiopulmonary exercise testing
Source: Front Cardiovasc Med. 2023 Feb 6;10:1098395. doi: 10.3389/fcvm.2023.1098395 (PMC9939638; doi:10.3389/fcvm.2023.1098395)
Supplement: Supplementary file 1 [file Table_1.docx]

**Supplementary Table 1:** **Baseline characteristics according to heart failure etiology stratified by sex**

|  | Normal | | | HFpEF | | | HFmrEF/HFrEF | | |
| --- | --- | --- | --- | --- | --- | --- | --- | --- | --- |
|  | Males | Females | P value | Males | Females | p value | Males | Females | p value |
| Age, years | 50(±18) | 58(±11) | 0.141 | 61(±18) | 62(±14) | 0.707 | 64(±15) | 63(±16) | 0.852 |
| Body surface area, m^2^ | 1.8(±0.14) | 1.72(±0.18) | **0.002** | 1.93(±0.18) | 1.73(±0.16) | **<0.001** | 1.98(±0.19) | 1.73(±0.25) | **<0.001** |
| Hemoglobin, g/dl | 14.2(±0.9) | 12.9(±2.4) | 0.244 | 13.2(±1.7) | 12(±1.8) | 0.065 | 13(±1.8) | 12.9(±2) | 0.875 |
| Heart rate, beats per minute | 75.2(±8.3) | 77.4(±10.3) | 0.233 | 78.7(±13.7) | 78.9(±15) | 0.947 | 79(±14.7) | 85.1(±19.2) | 0.166 |
| Systolic blood pressure, mmHg | 136(±23) | 142(±25) | 0.455 | 127(±52) | 143(±29) | 0.127 | 127(±35) | 135(±26) | 0.425 |
| Diastolic blood pressure, mmHg | 76(±21) | 85(±12) | 0.182 | 68(±30) | 79(±13) | 0.071 | 75(±18) | 83(±12) | 0.120 |
| Left ventricle end diastolic diameter, mm | 51(±7) | 44.1(±3.2) | **0.003** | 47.9(±6.5) | 45.11(±4.2) | **0.020** | 55.7(±9.6) | 51(±7.8) | 0.060 |
| Left ventricle end systolic diameter, mm | 34.1(±8.9) | 25.6(±3.4) | **0.005** | 30.6(±6.9) | 27.4(±5) | **0.030** | 42.1(±11.6) | 35.5(±11.5) | **0.040** |
| Left ventricle ejection fraction, % | 64(±16) | 61(±13) | 0.135 | 64.6(±9.6) | 63.9(±8.6) | 0.726 | 36.47(±9.1) | 37.33(±8.9) | 0.720 |
| Stroke volume index, ml/m^2^ | 40.4(±10.6) | 43.6(±9.2) | 0.437 | 38.77±7.3) | 38.1(±10.1) | 0.764 | 33.4(±9.2) | 37.3(±11.6) | 0.153 |
| Cardiac output, ml/min | 5.76(±1.86) | 5.6(±1.1) | 0.399 | 5.81(±1.36) | 5.19(±1.56) | **0.035** | 5.17(±1.69) | 5.16(±1.26) | 0.494 |
| Cardiac index, ml/min/m^2^ | 3.06(±0.96) | 3.4(±0.73) | 0.164 | 3.03(±0.75) | 3.03(±0.93) | 0.968 | 2.59(±0.72) | 3.0(±0.77) | **0.040** |
| Left ventricle end diastolic volume index, ml | 85(±37) | 75(±14) | 0.072 | 63.9(±17.8) | 60.7(±14.6) | 0.396 | 91(±32) | 73(±26) | **0.030** |
| Left ventricle end systolic volume index, ml | 40(±27) | 36(±12) | 0.068 | 22(±9.7) | 21(±7) | 0.304 | 59(±25) | 45(±17) | **0.030** |
| Left ventricle mass index, gr/m^2^ | 109(±31) | 86(±16) | 0.050 | 116(±41) | 98(±28) | 0.050 | 133(±38) | 107(±30) | 0.050 |
| Left atrial diameter, mm | 39(±9) | 38(±7) | 0.389 | 41(±8) | 43(±10) | 0.465 | 41(±14) | 43(±7) | 0.547 |
| Left atrial volume index, ml/m^2^ | 29.7(±12) | 32.6(±14.1) | 0.050 | 38.4(±18.3) | 54.7(±38.9) | **0.030** | 41.4(±20.5) | 40.8(±15.4) | 0.902 |
| E Wave cm/s base | 80.4(±38.8) | 91.1(±39.8) | 0.492 | 84.7(±34.3) | 117.6(±55.6) | **0.004** | 86.2(±23.1) | 97.9(±54.9) | **0.038** |
| A Wave cm/s base | 58.6(±31.7) | 71.7(±34.2) | 0.314 | 66.49(±27.2) | 72.4(±41.2) | 0.480 | 72.7(±28.4) | 81.47(±41.3) | 0.081 |
| Deceleration Time, ms | 234(±106) | 259(±136) | 0.580 | 231(±117) | 329(±205) | **0.010** | 200(±56) | 221(±106) | 0.338 |
| E' cm/s | 8.2(±2.7) | 8.1(±2.8) | 0.890 | 5.9(±2.16) | 5.7(±2.5) | 0.684 | 5.6(±2.3) | 5.6(±1.7) | 0.727 |
| E/e' | 11.1(±9.2) | 13.3(±10) | 0.574 | 15.68(±3.9) | 24.4(±11.8) | **0.010** | 17.2(±12.7) | 20.6(±13.9) | 0.356 |
| Systolic pulmonary artery pressure, mmHg | 28.9(±5.92) | 28.5(±5.83) | 0.888 | 34.9(±11.6) | 36(±14.7) | 0.781 | 34.8(±11.4) | 38.6(±10.4) | 0.339 |
| S wave, cm/s | 7.1(±1.5) | 6.1(±1.2) | 0.138 | 5.64(±1.94) | 4.9(±1.5) | 0.131 | 5.2(±2.1) | 4.5(±1.2) | 0.151 |
| Right atrial area, cm^2^ | 15.4(±2.6) | 13.8(±5) | 0.139 | 17.2(±5.1) | 16(±6.9) | 0.326 | 19.5(±7.2) | 15.1(±4.9) | **0.010** |
| Right ventricle end diastolic area, cm^2^ | 26.5(±7.3) | 21.3(±5.5) | 0.058 | 21.1(±5) | 18.6(±3.6) | **0.010** | 27(±7.6) | 20.7(±5.8) | **0.003** |
| Right ventricle end systolic area, cm^2^ | 15.2(±3.8) | 12.8(±5.2) | 0.085 | 12.3(±3.4) | 10.6(±2.9) | **0.030** | 17(±6.2) | 13.4(±4.3) | **0.020** |
| Right ventricle fractional area change, % | 0.41(±0.1) | 0.4(±0.13) | 0.060 | 0.42(±0.11) | 0.43(±0.12) | 0.753 | 0.36(±0.11) | 0.35(±0.12) | 0.893 |
| FEV1, % Predicted | 97.0(±12.9) | 97.3(±12.7) | **0.031** | 85.2(±19.2) | 79.5(±18.2) | 0.190 | 85.3(±17.5) | 84.4(±18.8) | 0.843 |
| FVC, % Predicted | 89.6(±14.4) | 85.6(±13.7) | 0.396 | 81.5(±19.4) | 81.2(±17) | 0.943 | 80.3(±17.9) | 87.2(±21.1) | 0.190 |
| FEV1-FVC, % Predicted | 112(±9.8) | 109(±10.5) | 0.361 | 110(±20) | 104(±12) | 0.121 | 109(±10) | 104(±9) | 0.089 |
| Peak VO_2_ | 2.5(±0.8) | 1.5(±0.6) | **0.0003** | 1.3(±0.4) | 0.95(±0.3) | **0.0002** | 1.2(±0.4) | 0.8(±0.2) | **<0.0001** |
| Peak VO_2_/kg | 32.3(±9.6) | 20.5(±6.8) | **0.0002** | 15.6(±5.6) | 13.9(±5.2) | 0.070 | 15.0(±4.9) | 12.3(±3.7) | **0.020** |

HFpEF=Heart failure preserved ejecrtion fraction; HF*mr*EF/HF*r*EF= Heart failure mildly reduced ejection fraction/Heart failure reduced ejection fraction
